# Pleasant and Unpleasant Odors Influence Hedonic Evaluations of Human Faces: An Event-Related Potential Study

**DOI:** 10.3389/fnhum.2015.00661

**Published:** 2015-12-16

**Authors:** Stephanie Cook, Nicholas Fallon, Hazel Wright, Anna Thomas, Timo Giesbrecht, Matt Field, Andrej Stancak

**Affiliations:** ^1^Department of Psychological Sciences, University of LiverpoolLiverpool, UK; ^2^Department of Research and Development, UnileverPort Sunlight, UK; ^3^Department of Research and Development, UnileverVlaardingen, Netherlands

**Keywords:** odors, faces, perception, evaluation, event-related-potential

## Abstract

Odors can alter hedonic evaluations of human faces, but the neural mechanisms of such effects are poorly understood. The present study aimed to analyze the neural underpinning of odor-induced changes in evaluations of human faces in an odor-priming paradigm, using event-related potentials (ERPs). Healthy, young participants (*N* = 20) rated neutral faces presented after a 3 s pulse of a pleasant odor (jasmine), unpleasant odor (methylmercaptan), or no-odor control (clean air). Neutral faces presented in the pleasant odor condition were rated more pleasant than the same faces presented in the no-odor control condition, which in turn were rated more pleasant than faces in the unpleasant odor condition. Analysis of face-related potentials revealed four clusters of electrodes significantly affected by odor condition at specific time points during long-latency epochs (600−950 ms). In the 620−640 ms interval, two scalp-time clusters showed greater negative potential in the right parietal electrodes in response to faces in the pleasant odor condition, compared to those in the no-odor and unpleasant odor conditions. At 926 ms, face-related potentials showed greater positivity in response to faces in the pleasant and unpleasant odor conditions at the left and right lateral frontal-temporal electrodes, respectively. Our data shows that odor-induced shifts in evaluations of faces were associated with amplitude changes in the late (>600) and ultra-late (>900 ms) latency epochs. The observed amplitude changes during the ultra-late epoch are consistent with a left/right hemisphere bias towards pleasant/unpleasant odor effects. Odors alter evaluations of human faces, even when there is a temporal lag between presentation of odors and faces. Our results provide an initial understanding of the neural mechanisms underlying effects of odors on hedonic evaluations.

## Introduction

A number of behavioral studies have investigated cross-modal effects of odors on evaluations of human faces (Todrank et al., 1995; Leppanen and Hietanen, 2003; Demattè et al., 2007; Li et al., 2007; Seubert et al., 2014). In general, pleasant odors increased preferences for faces, with unpleasant odors having the opposite effect. The neural mechanisms that underlie such effects are not yet established. One study found that repeated pairing of emotionally neutral faces with pleasant and unpleasant odors resulted in conditioned shifts in face ratings (when presented subsequently, without odors), but failed to show any significant cortical changes related to conditioning (Hermann et al., 2000). Another study paired pleasant and unpleasant odors with positively and negatively valenced facial expressions, demonstrating evaluative changes that occurred as a function of hedonic congruency between the odor-prime and target-face and increased late-positive potential (LPP) amplitude for incongruent odor-face pairings (Bensafi et al., 2002a). However, neural processes underlying immediate odor-induced changes in evaluations of emotionally neutral faces, where evaluative congruency or conditioned pairing do not play a role, remain unknown.

Most previous studies investigating effects of odors on immediate evaluations of faces used paradigms where the odor-primes and target-faces overlapped (Leppanen and Hietanen, 2003; Demattè et al., 2007; Seubert et al., 2014), or where target-faces appeared at the offset of the odor-prime (Bensafi et al., 2002a). This complicates interpretation of the findings, because any shift in target evaluation could be attributable to affective responses to the odors themselves. It is important to establish whether or not odor-related evaluative shifts can survive after inserting a temporal lag between odor-primes and target-faces. This should ensure unbiased shifts in evaluative ratings that occur as a result of priming effects activated by the odor valences, which then carry over to the evaluation of the target-face.

The aim of the present study was to investigate the neural underpinning of odor-induced changes in immediate hedonic evaluations of neutral faces, by observing the influence of both pleasant and unpleasant odors on evaluations of emotionally neutral male and female faces that were presented 1 s after odor offset. We used a novel and exploratory approach to analyze odor-induced modulations in the ERP response to faces. Based on the previous literature, we hypothesized that faces in the pleasant odor condition would be rated as most pleasant, faces in the unpleasant odor condition would be rated as least pleasant, and faces in the clean air condition would be rated in between the two. We also hypothesized that odor-induced change in the ERP response to faces would be reflected in the LPP.

## Materials and Methods

### Participants

A total of 23 (11 male) participants aged 18−36 years (mean ± standard deviation: 24.65 ± 4.35) were screened in a session prior to the experiment after responding to the study advertisement. All but four participants were right-handed. People suffering from asthma or neurological disorders, particularly anosmia or epilepsy, were not permitted to take part in the study. Normal olfactory function was ascertained using the Sniffin’Sticks (Hummel et al., 1997) test battery. Participants had to successfully identify a minimum of 9 out of the 12 odors in order to take part in the experiment. The mean score on the Sniffin’Sticks odor identification task was 10.5 (± 1.5). Three people were excluded from participation at the screening stage after scoring below nine on the Sniffin’Sticks task. Hence, a total of 20 participants (mean age: 25.15 ± 4.43) participated in the experiment. Participants were asked not to smoke, drink coffee or chew gum for 2 h prior to the experiment, and were asked to minimize their use of fragranced products on the day. Participants were reimbursed for their time and travel expenses. The study was approved by the Research Ethics Committee at the University of Liverpool. All subjects gave written informed consent in accordance with the Declaration of Helsinki.

### Visual and Olfactory Stimuli

A total of 36 (18 male) neutral faces obtained from the NimStim Set of Facial Expressions (Tottenham et al., 2009) were used in the experiment. Out of the 18 female faces, nine were white/Caucasian, five were East-Asian, and four were Afro-Caribbean. Out of the 18 male faces, 12 were white/Caucasian, five were afro-Caribbean and one was East-Asian. Participants were gathered from a student population at the University of Liverpool, and were therefore a mixture of races and ethnicities with a white/Caucasian majority. Data on the race/ethnicity of participants was not recorded for ethical reasons. All face images were frontal views, in color, with a consistent light background. All images measured 253 × 312 pixels. During the screening session, participants rated the perceived pleasantness of the facial expressions of all 36 faces (on a scale ranging from 0-very unpleasant to 100-very pleasant) in order to ensure that they were perceived as neutral. The mean face pleasantness rating was 47.80 (± 7.2).

Odors were administered through two tubes approximately 2 cm away from the nostrils; using a custom-built, continuous airflow, computer-controlled olfactometer with eight channels (Dancer Design Ltd., UK). Odor pulses were embedded within a constant flow of clean air, in order to avoid effects of a sudden increase in airflow associated with presentation of an odor (Huart et al., 2012). Airflow was kept constant at approximately 2.2 l/min.

There were three odor conditions in the experiment; pleasant, unpleasant and a neutral control. Methylmercaptan (1% dilution in Propylene Glycol), a rotten cabbage-like odor, was selected for the unpleasant condition. Jasmine odor (no dilution) was selected for the pleasant condition. These dilutions were matched on perceived intensity based on data from a pilot study carried out on a separate sample prior to the experiment (*N* = 15). Odors were supplied by Symrise Ltd. (Netherlands). Propylene Glycol (1,2-Propanediol 99%, Sigma-Aldrich Ltd., UK) was used for dilution, the clean air control and constant flow.

Both presentation of the visual task stimuli and triggering of the odor valves was accomplished using Cogent software for Matlab (MATLAB v. R2011a program, The MathWorks, Inc., USA). In between experimental blocks and sessions, a Blueair 203 air purifier (Blueair Ltd., Sweden) was used to minimize any residual odor that may have carried into the next experimental block or session.

### Recordings

EEG was recorded continuously using a 129-channel Geodesics EGI System (Electrical Geodesics, Inc., Eugene, OR, USA) with the sponge-based Geodesic Sensor Net. The sensor net was aligned with respect to three anatomical landmarks; two pre-auricular points and the naison. Electrode-to-skin impedances were kept below 50 kΩ and at equal levels across all electrodes. The recording band-pass filter was 0.01−1000 Hz, and the sampling rate was 1000 Hz. The electrode Cz was used as the reference.

Participants’ respiration and pulse rate was recorded continuously throughout the experiment with a piezoelectric respiratory belt transducer worn around the chest at the level of the epigastrium, and a finger pulse oximeter transducer worn on the index finger of the left hand (ADInstruments Ltd., Oxford, UK). Signals were transduced and extracted using LabChart 7 (ADInstruments Ltd., Oxford, UK).

### Procedure

After application of the EEG cap, participants were led into a dimly lit, sound attenuated room and sat facing a 19 inch CRT monitor (60 Hz refresh rate) placed 0.7 m in front of them. First, the respiratory and pulse monitoring equipment was fitted onto participants and the signals were checked. Following this, the olfactometer head piece was fitted, and participants were given some instructions. The experimental session lasted around 1 h in total, including baseline odor ratings and the experimental task. Ratings of odor pleasantness, intensity and familiarity were recorded before and after the task. Odors were administered individually, in a 4 s pulse manually triggered to coincide with the onset of inspiration. After each odor pulse, on-screen visual analog scales prompted participants to rate the pleasantness (from 0-very unpleasant to 100-very pleasant), intensity (0-no odor to 100-very intense odor) and familiarity (0-not familiar at all to 100-extremely familiar) of the odor.

**Figure 1:**
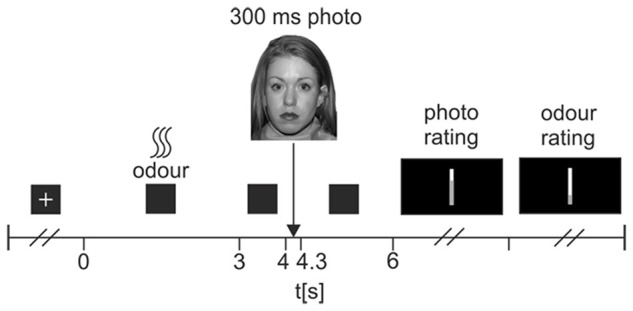
**Flowchart of experimental trial procedure.** At the start of each trial, participants viewed a white fixation cross on a black background. Participants were instructed to relax and breathe normally during this time. At the very onset of a participant’s inspiration, the experimenter triggered a 3 s odor pulse, which was followed then by a 1 s pause where participants viewed a black screen. Following this, a photograph of a neutral face was displayed for 300 ms, followed by a 1700 ms rest period where participants viewed a black screen. A visual-analog scale then prompted participants to rate the pleasantness of the photograph (very unpleasant—very pleasant). A second scale then prompted participants to rate the intensity of the odor (no odor—very intense odor). Once participants had completed both ratings, the next trial began.

The experimental task was split into three blocks of 36 trials. Trials were pseudo-randomly ordered, such that each of the 36 faces used in the task appeared only once in each block, and once with each odor. Odor presentation was also pseudo-random, such that all three odors were presented across all three blocks, but no two consecutive trials used the same odor. Figure 1 shows a flowchart of the trial procedure. Each trial began with a resting interval during which subjects viewed a white cross on a black background. Duration of this interval was dependent upon the triggering of the odor pulse; the experimenter observed participants’ respiratory waveforms, and manually triggered the odor pulses at the very onset of inspiration. A 3 s odor pulse was then released, during which participants viewed a black screen. The screen remained black for a further 1 s resting interval, before a neutral face was displayed on-screen for 300 ms. Following this, a 1700 ms resting interval with a black screen preceded a rating scale prompting participants to rate the pleasantness of the neutral face (from 0-very unpleasant to 100-very pleasant). Once participants had responded, a second scale prompted them to rate the intensity of the odor administered in that trial (0-no odor to 100-very intense odor). After their response, the next trial began.

### Behavioral Analysis

Ratings of odor pleasantness, intensity and familiarity were analyzed using 3 × 2 repeated measures ANOVAs. The independent variables were odor condition (clean air, methylmercaptan and jasmine), and time (before/after priming task). Data from the experimental task were analyzed using one-way ANOVAs, observing differences in face pleasantness ratings and odor intensity ratings across the three odor conditions. Two-way ANOVAs were used to investigate effects of gender and experimental block on face pleasantness and odor intensity ratings. Significant main effects were investigated using pairwise comparisons; significant interactions were followed up with *post hoc* *t*-tests. All behavioral data were analyzed using SPSS v. 22 software package (IBM Inc., USA).

### ERP Analysis

EEG recordings were pre-processed using BESA v. 6.0 (MEGIS GmbH, Germany). Data were first referenced to a common average using common averaging method (Lehmann, 1987). The oculographic and when necessary, electrocardiographic artifacts were removed by principal component analysis (Berg and Scherg, 1994). Data were visually inspected for the presence of any movement or muscle artifacts, and epochs contaminated with artifacts were excluded. The average numbers of accepted trials in each condition were as follows: clean air, 33.75 (± 2.07); jasmine, 33.65 (± 1.75); methylmercaptan, 32.9 (± 1.68). The average number of trials accepted did not differ across conditions (*p* > 0.05).

Data were band-pass filtered from 0.5−30 Hz and down-sampled to a rate of 256 Hz, and exported from BESA into the SPM 12 software package (Statistical Parametric Mapping, UCL, England; http://www.fil.ion.ucl.ac.uk/spm/software/spm12/). Event-related potentials (ERPs) in response to neutral faces were computed separately for each odor condition by averaging respective epochs in the intervals ranging from 300 ms before photo onset to 1200 ms after photo onset. The baseline period ranged from −300 to 0 ms relative to the onset of the visual stimulus. Grand average waveforms were computed.

Effects of odors on face processing have been shown to span over multiple ERP components (Cacioppo et al., 1993; Bentin et al., 1996; Rossion and Jacques, 2001; Hajcak et al., 2006, 2007; Duval et al., 2013). Further, relatively subtle effects of odors on hedonic aspects of face perception would likely involve late potential components, such as the LPP known to operate in a long latency window from 600−2000 ms (Cacioppo et al., 1993; Cuthbert et al., 2000; Hajcak et al., 2006, 2007, 2010; Hajcak and Olvet, 2008; MacNamara and Hajcak, 2010; Weinberg and Hajcak, 2010; Duval et al., 2013). The late potential components do not show a distinct potential peak allowing for a traditional ERP analysis in which ERP data would be reduced to only a small number of components based on their peak latencies. Therefore, we applied an omnibus analysis of effects of odors on ERPs involving all time points from 0 to 1000 ms and all scalp sites, allowing us to explore effects of odors on ERPs without applying* a priori* knowledge of peak latencies. The Statistical Parametric Mapping (SPM) software combines advanced statistical models with robust control for Type I error (Poline et al., 1997; Kiebel and Friston, 2004). In contrast to alternative approaches, such as permutation analysis of clusters of ERPs over the epoch time (Maris and Oostenveld, 2007), SPM applies the theory of random fields to the volumes of space-time data which allows to evaluate the degrees of freedom in evaluation of statistical test results based on the spatial and temporal complexity of data (Worsley, 2003).

The statistical analysis was performed in two steps. In the initial exploratory step, EEG data were converted into three-dimensional scalp-time images using SPM. The electrodes were mapped onto a standardized scalp grid sized 32 × 32 pixels (pixel size 4.25 × 5.3 mm^2^), representing the field potential planes stacked over the time axis. Images were smoothed with a Gaussian kernel of 9 × 9 × 20 mm^2^·ms (full width at half maximum). Data from over the whole epoch (385 time samples) and all standardized scalp points were screened for a statistically significant effect of odors using a one-way ANOVA for repeated measures. We applied a liberal, uncorrected threshold of *p* = 0.005 and the cluster size of 20 contiguous space-time voxels to detect clusters affected by odors. The amplitude data from these clusters were subsequently analyzed using further one-way ANOVA for repeated measures in SPSS v. 22 (IBM Inc., USA). The statistical threshold of this confirmatory analysis was *p* = 0.05.

### Analysis of Respiratory Movements

Respiratory signals were low-pass filtered and averaged separately for each of the three odor conditions, then analyzed statistically using a one-way ANOVA in Matlab. The 10 s analysis window ranged from 3 s before to 7 s after onset of odor, with the interval 7–8 s overlapping with the ERP analysis epoch. A permutation analysis with 2000 permutations was used to correct the *P* values. We used a one-way ANCOVA for repeated measures in BMDP 2V program (Biomedical Data Package, Cork, Ireland) to analyze whether changes in respiratory movement patterns contributed to the effects of odors seen in ERP clusters.

## Results

### Odor Ratings

Table 1 shows the mean ratings of odor pleasantness, intensity and familiarity before (Time 1) and after (Time 2) the priming task. A repeated-measures ANOVA confirmed a significant main effect of odor type on pleasantness ratings across both time points (*F*_(2,38)_ = 95.93, ${\text{η}}_{p}^{2}$ = 0.84, *p* < 0.001). Overall, jasmine was rated as most pleasant (76.20 ± 16.6), methylmercaptan as least pleasant (12.31 ± 15.37), and clean air was rated close to neutral (55.22 ± 10.78). Pairwise comparisons indicated that all three odors significantly differed from each other in terms of pleasantness (*p* < 0.001). There was no main effect of time (before/after task), or interaction between time and odor affecting pleasantness ratings (*p* > 0.05), suggesting that perceptions of odor pleasantness remained stable throughout the experiment.

**Table 1:** **Mean baseline ratings of pleasantness, intensity and familiarity ratings of the three odors taken before and after the task**.

	Pleasantness		Intensity		Familiarity	
	Time 1	Time 2	Time 1	Time 2	Time 1	Time 2
Clean Air	54.07 (± 8.16)	56.36 (± 13.23)	13.02 (± 19.5)	2.27 (± 3.8)	80.72 (± 23.53)	84.95 (± 17.94)
Jasmine	74.7 (± 13.15)	77.72 (± 20.05)	62.8 (± 16.27)	74.31 (± 15.91)	63.82 (± 24.61)	72.25 (± 24.3)
Methylmercaptan	13.5 (± 14.12)	11.13 (± 17.19)	84.95 (± 8.4)	83.21 (± 15.44)	52.3 (± 29.5)	61.8 (± 32.93)

A repeated-measures ANOVA revealed a significant main effect of odor on intensity ratings across both time points (*F*_(2,38)_ = 318.41, ${\text{η}}_{p}^{2}$ = 0.94, *p* < 0.001). Pairwise comparisons indicated that jasmine was perceived as significantly more intense (68.6 ± 16.71) than clean air (7.64 ± 14.7; *p* < 0.001). In spite of pilot data suggesting that the jasmine and methylmercaptan odors were matched for perceived intensity, pairwise comparisons showed that methylmercaptan was perceived as significantly more intense (84.08 ± 12.2) than both jasmine (*p* < 0.001) and clean air (*p* < 0.001) across both time points. There was no main effect of time on intensity ratings; however there was an interaction between time and odor showing effects on intensity ratings (*F*_(2,38)_ = 10.18, ${\text{η}}_{p}^{2}$ = 0.35, *p* < 0.001). *Post hoc* *t*-tests were employed to further investigate this interaction. These confirmed that clean air was perceived as less intense at Time 2 (after the priming task) in comparison to Time 1 (before the priming task; *t*_(19)_ = 2.61, *p* = 0.02). Further, jasmine was perceived as more intense at Time 2 in comparison to Time 1 (*t*_(19)_ = −2.83, *p* = 0.01). There was no significant difference in intensity ratings of methylmercaptan across time points (*p* > 0.05).

A repeated-measures ANOVA confirmed a significant main effect of odor on familiarity ratings across both time points (*F*_(2,38)_ = 7.91, ${\text{η}}_{p}^{2}$ = 0.29, *p* = 0.001). Pairwise comparisons indicated that clean air was rated as more familiar (82.83 ± 20.51) than both jasmine (68.03 ± 24.20; *p* = 0.02), and methylmercaptan (57.04 ± 30.82; *p* = 0.004). There was no difference in familiarity ratings of jasmine and methylmercaptan (*p* > 0.05), and there was no main effect of time, or interaction between time and odor affecting familiarity ratings (*p* > 0.05).

### Face and Odor Ratings During the Experiment

Table 2 shows the mean pleasantness ratings of faces under each odor condition. A one-way ANOVA revealed a significant effect of odor on pleasantness ratings of faces (*F*_(2,38)_ = 13.41, ${\text{η}}_{p}^{2}$ = 0.41, *p* < 0.001). Pairwise comparisons indicated that neutral faces were rated as more pleasant after presentation of the jasmine odor in comparison to faces in both the clean air (*t*_(19)_ = 3, *p* = 0.007) and methylmercaptan (*t*_(19)_ = 4.16, *p* = 0.001) conditions; and faces in the methylmercaptan condition were rated as significantly less pleasant than those in the clean air condition (*t*_(19)_ = −3.09, *p* = 0.006).

**Table 2:** **Mean pleasantness ratings of neutral face photographs and odor intensity ratings under three odor conditions during the experimental task**.

	Face rating	Odor intensity rating
Clean Air	53.19 (± 4.1)	5.6 (± 7.05)
Jasmine	55.26 (± 4.3)	56.33 (± 15.83)
Methylmercaptan	50.19 (± 3.92)	61.34 (± 17.68)

We analyzed whether odors affected pleasantness ratings of faces differently in male and female participants. A two-way mixed ANOVA (male vs. female participants, three odors) showed no significant effect of participant gender on face ratings (*p* > 0.05). Importantly, there was no significant interaction between odor and gender affecting face ratings (*p* > 0.05), and therefore data were analyzed further without splitting them based on the gender factor.

We also evaluated effects of experimental block on effects of odors on face pleasantness ratings. The statistical analysis consisted of two-way ANOVAs with three odors and three experimental blocks as independent variables. There was an interaction between odor and block affecting face pleasantness ratings (*F*_(4,76)_ = 4.95, ${\text{η}}_{p}^{2}$ = 0.2, *p* = 0.003). *Post hoc* one-way ANOVAs showed that in the pleasant odor condition, there was a significant effect of block (*F*_(2,38)_ = 5.27, ${\text{η}}_{p}^{2}$ = 0.22, *p* = 0.14), with pairwise comparisons indicating that faces presented in the pleasant odor condition were rated as more pleasant in block 2 of the experiment in comparison to both block 1 (*p* = 0.05) and block 3 (*p* = 0.001). In the unpleasant odor condition, the effect of block was statistically significant (*F*_(2,38)_ = 6.15, ${\text{η}}_{p}^{2}$ = 0.25, *p* = 0.006). Pairwise comparisons indicated that faces presented in the unpleasant odor condition were rated as less pleasant in blocks 2 and 3 in comparison to when they were presented in block 1 (*p* = 0.008 and *p* = 0.017, respectively).

Table 2 also shows the mean odor intensity ratings for each odor condition. A one-way ANOVA revealed a significant effect of odor on intensity ratings (*F*_(2,38)_ = 180.74, ${\text{η}}_{p}^{2}$ = 0.91, *p* < 0.001). Pairwise comparisons indicated that both jasmine (*t*_(19)_ = −15.51, *p* < 0.001) and methylmercaptan (*t*_(19)_ = −14.34, *p* < 0.001) were rated as significantly more intense than clean air. There was no significant difference between intensity ratings of jasmine and methylmercaptan (*t*_(19)_ = −2.08, *p* > 0.05).

Odor intensity ratings also changed over the course of the experiment (*F*_(2,38)_ = 11.62, ${\text{η}}_{p}^{2}$ = 0.38, *p* = 0.001). Pairwise comparisons indicated that all odors were rated as most intense during block 1 (mean ± SE 45.42 ± 2.16), and least intense during block 3 (37.44 ± 2.87). Odors in block 2 were rated in between the two (40.05 ± 2.91; *p* < 0.05). However, there was no significant interaction between odor and block affecting odor intensity ratings (*p* > 0.05).

### ERP Components

**Figure 2:**
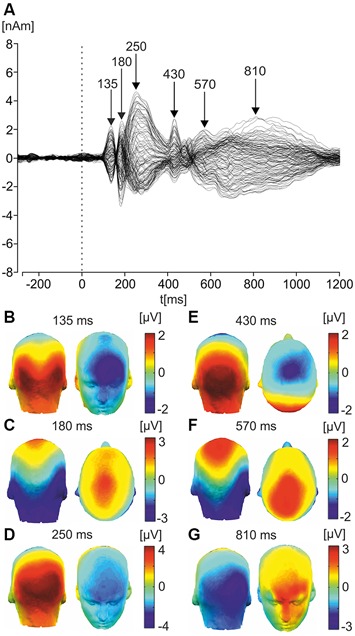
**Butterfly plot of grand average ERP response to faces and corresponding scalp topographies. (A)** Butterfly plot of grand average ERPs in response to faces. Peak latencies of distinct ERP components (135, 180, 250, 430, 570, and 810 ms) are highlighted with arrows. **(B)** Latency component 135 ms (P1). The topographic maps of grand average ERPs overlaid on the volume rendering of the human head are shown. **(C)** Latency component 180 ms (N170). **(D)** Latency component 250 ms (P300). **(E)** Latency component 430 ms (N400). **(F)** Latency component 570 ms (late component/LPP). **(G)** Latency component 810 ms (ultra-late component/LPP).

Figure 2 illustrates the ERPs in response to faces across all trials and all odor conditions in the form of a butterfly plot and topographic maps of selected potential components. Topography of the first component showed bilateral positivity over the occipital electrodes and negativity over frontal electrodes, peaking around 135 ms, consistent with characteristics of the P1 component-related to early processing of visual stimuli (Hopf et al., 2002). Further, the second component, peaking around 175 ms, showed strong negativity over posterior parietal and temporal electrodes, consistent with characteristics of the N170 face-processing component (Bentin et al., 1996).

The next component peaked around 250 ms, showing strong positivity over occipital/parietal electrode sites, consistent with the P300 component, which is involved in information-processing in attentional and memory mechanisms (Polich, 2012). The fourth component was similar, peaking at approximately 430 ms and showing negativity over centro-parietal electrode sites; consistent with the N400 component, which is implicated in the processing of meaningful stimuli, including faces (Kutas and Federmeier, 2011).

A further component was a long component beginning around 500 ms and peaking at approximately 570 ms. Showing negativity over occipital electrode sites and positivity over parietal areas, it had a similar topography to the N170 and was consistent with characteristics of the LPP which is sensitive to the emotional content of pictures, words and faces (Cacioppo et al., 1993; Cuthbert et al., 2000; Hajcak et al., 2006, 2007). The final component was a second long-latency component, beginning around 650 ms and extending until 1000 ms, it peaked around 810 ms and showed negativity over the right temporal-parietal electrodes, and positivity over frontal electrodes. These two late components are comparable with the mid- and late-LPP components observed in a recent study investigating ERPs in response to faces (Duval et al., 2013).

### Effects of Odors on ERPs

**Figure 3:**
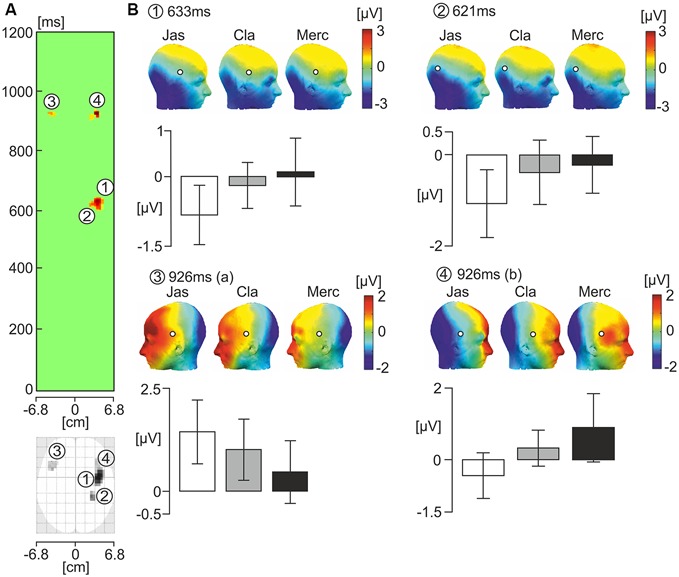
**One-way ANOVA showing the effects of the three odor conditions on ERP response to faces. (A)** The green panel shows statistically significant latency periods (uncorrected *p* < 0.005) in the scalp-time plot where F values represent the strength of variance between odor conditions over the horizontal axis of the scalp in every time sample from 0 and 1200 ms relative to the onset of the face photograph. The scalp values over the horizontal axis of the scalp are averages of F values occurring at each vertical point for a given horizontal point in the standardized scalp map (from −6.8 to +6.8 cm). Two latency intervals showed the presence of statistically significant spatio-temporal clusters. In the interval 600−640 ms, two clusters numbered 1 and 2 showed a significant effect of odor condition. In the latency period 910−930 ms, clusters numbered 3 and 4 showed a significant effect of odor condition. Below the green panel is the standard scalp map of statistically significant clusters using ERPs. **(B)** Corresponding topographic maps of the numbered significant cluster latencies under each odor condition (Jas-jasmine, pleasant odor; Cla-clean air, control; Merc-methylmercaptan, unpleasant odor). White circles with a black outline pinpoint the location of the significant electrode clusters. Bar graphs below illustrate the mean EEG amplitude for each cluster/latency under each odor condition (μV). White bars represent the pleasant odor condition, gray bars represent the neutral control condition, and black bars represent the unpleasant odor condition.

SPM 12 software was used to compute a one-way ANOVA on smoothed scalp-time images of data from 0−1000 ms relative to onset of the face. The one-way ANOVA revealed four scalp-time clusters of that showed significant effects of odor. Amplitude data from each of these scalp-time clusters was then extracted, and further one-way ANOVAs were computed on the data using SPSS. Figure 3 illustrates these significant scalp-time clusters. The corresponding topographic maps from each odor condition for each significant cluster are shown with bar graphs showing the mean EEG scalp-amplitude (μV).

At 621 and 633 ms following onset of the face photograph, there was a significant effect of odor in the right parietal electrodes. Given that the two clusters were within 20 ms of one another, it is likely that they reflect a similar process. In a preliminary analysis, we analyzed the amplitude data from these two clusters in a two-way ANOVA, with odor and cluster as independent variables. There was no significant effect of cluster, or interaction between odor and cluster affecting amplitude (*p* > 0.05). Therefore, we chose to average the amplitude data from the two clusters. There was a significant effect of odor on the averaged amplitude data from clusters at 621 and 633 ms following onset of the face (*F*_(2,38)_ = 7.89, ${\text{η}}_{p}^{2}$ = 0.29, *p* = 0.001). Pairwise comparisons indicated a significantly stronger negative amplitude for faces presented after administration of the jasmine odor in comparison to those in both the clean air (*p* = 0.01) and methylmercaptan (*p* = 0.001) conditions. There was no significant difference in amplitude between the clean air and methylmercaptan conditions (*p* > 0.05).

At 926 ms following the onset of the face, there were two significant clusters; one in the left hemisphere (*F*_(2,38)_ = 4.84, ${\text{η}}_{p}^{2}$ = 0.2, *p* = 0.014), and one in the right hemisphere (*F*_(2,38)_ = 4.72, ${\text{η}}_{p}^{2}$ = 0.2, *p* = 0.026), both at lateral fronto-temporal electrode sites. Pairwise comparisons indicated that in the left hemisphere, the positive amplitude was significantly greater in the jasmine condition compared to the methylmercaptan condition (*p* = 0.003). Amplitude differences between the jasmine and clean air, and clean air and methylmercaptan conditions were non-significant (*p* > 0.05). In the right hemisphere, there was significantly greater positive amplitude in the methylmercaptan condition in comparison to the jasmine condition (*p* = 0.009). The amplitude difference between the clean air and jasmine conditions was also significant (*p* = 0.02), but there was no significant difference in amplitude between the clean air and methylmercaptan conditions (*p* > 0.05).

Pearson correlation analyses were computed with amplitude data from each significant scalp-time cluster (621, 633 and 926 ms-left and right hemisphere), baseline odor pleasantness and intensity ratings (taken before and after the task), face ratings throughout the task, and odor intensity ratings throughout the task, for both pleasant and unpleasant odor conditions. Table 3 shows Pearson’s *r* correlation coefficients and statistical values for bivariate correlations between amplitude data and subjective ratings. Of these correlations, one remained significant after applying Bonferroni-Šidák correction for multiple comparisons. Odor pleasantness ratings, and left hemisphere potential amplitude at 926 ms in the unpleasant odor condition were negatively correlated (*r*_(20)_ = −0.62, *p* = 0.003). Baseline intensity ratings and left-hemisphere amplitude at 926 ms in the pleasant odor condition were positively correlated, but only borderline significant after Bonferroni-Šidák correction, (*r*_(20)_ = 0.56, *p* = 0.01). No correlations between amplitude data and photo/odor ratings throughout the task reached significance (*p* > 0.05).

**Table 3:** **Pearson correlations (*r* and *P* values) for amplitude data at each significant scalp-time cluster and baseline ratings of odor pleasantness and intensity, photo ratings and odor intensity ratings throughout the task for both pleasant and unpleasant odor conditions**.

Pleasant odor								
Cluster	Baseline pleasantness		Baseline intensity		Photo rating		Odor rating	
	*r*	*P*	*r*	*P*	*r*	*P*	*r*	*P*
621 ms	−0.22	0.36	0.05	0.84	0.35	0.13	−0.34	0.14
633 ms	−0.42	0.86	0.11	0.64	0.02	0.93	0.06	0.81
926 ms^a^	0.39	0.08	0.56	0.01*	−0.28	0.23	−0.05	0.84
926 ms^b^	−0.41	0.07	−0.13	0.59	0.23	0.34	0.06	0.79

Unpleasant odor								

621 ms	−0.11	0.65	−0.01	0.99	−0.15	0.52	−0.83	0.73
633 ms	−0.12	0.61	0.07	0.76	−0.19	0.42	−0.01	0.99
926 ms^a^	−0.62	0.003**	0.11	0.65	0.35	0.13	−0.04	0.86
926 ms^b^	0.19	0.41	−0.09	0.68	−0.05	0.85	−0.22	0.35

Correlation is significant at the p < 0.05 level (two-tailed) following Bonferroni- Šidák correction for multiple tests. Correlation marked * was significant at p < 0.05, correlation marked ** was significant at the p < 0.05 level following Bonferroni-Šidák correction. 926 ms^a^ and 926 ms^b^ represent clusters at 926 ms in the left and right hemispheres, respectively.

### Analysis of Respiratory Data

**Figure 4:**
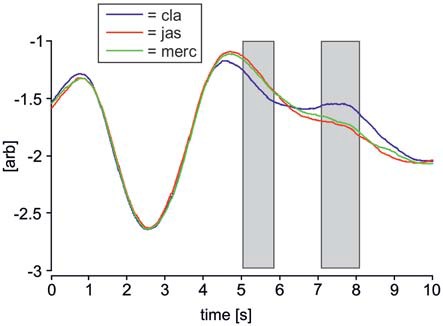
**Average respiratory waveforms for each odor condition.** Respiratory movement signals from every subject across all trials were averaged over a period of 10 s, beginning 3 s prior to odor onset. Time 3 represents odor onset, time 7 represents onset of the visual face stimulus. The blue line represents clean air trials (denoted as “cla”), the red line represents pleasant odor trials (“jas”) and the green line represents unpleasant odor trials (“merc”). Two gray rectangles indicate time intervals in which the three respiratory movement signals differed significantly according to a one-way ANOVA for repeated measures (*p* < 0.05). Upwards deflections of respiratory signals corresponds to inspiration.

Figure 4 shows averaged respiratory waveforms for each odor condition in a 10 s interval, beginning 3 s prior to odor onset. Odors significantly affected respiratory movements in two intervals, one 5–5.8 s, and another 7.1–8.1 s. The latter interval overlapped with the period in which ERPs were recorded and analyzed. In both intervals showing a statistically significant effect of odors, the respiratory movements in the clean air condition differed from both pleasant and unpleasant odor conditions. However, a one-way ANCOVA for repeated measures showed that there were no statistically significant covariate effects of respiratory movements on ERP data from any of the four clusters (621, 633, 926 ms, left and right hemisphere; *p* > 0.05). Therefore, it is unlikely that differences in respiratory movements affected odor-related ERP changes.

## Discussion

Our study was the first to investigate effects of pleasant and unpleasant odors on evaluations of neutral male and female faces using a novel approach to ERP analysis. We analyzed ERP data from all electrodes across all time points relative to onset of the faces, to begin to provide an understanding of the processes that might underlie odor-related evaluative shifts during face perception. Behavioral data revealed the predicted effects of odors on face ratings: neutral faces preceded by a pleasant odor prime were rated most pleasant, whereas those preceded by an unpleasant odor prime were rated as least pleasant. Faces presented in the clean air control condition were rated in between the two. ERP data revealed that odors modulated amplitudes of late and ultra-late event-related potential components from 600–950 ms. Topographic maps showed greater negativity in the right posterior- and temporal-parietal electrodes in response to faces in the pleasant odor condition in clusters at 621 and 633 ms. At 926 ms, topographies indicated greater positivity in response to faces in the pleasant and unpleasant odor conditions in the left and right lateral fronto-temporal electrodes, respectively.

The behavioral data are consistent with previous findings that odors shift hedonic evaluations of faces (Todrank et al., 1995; Hermann et al., 2000; Leppanen and Hietanen, 2003; Demattè et al., 2007; Li et al., 2007; Seubert et al., 2014). The inclusion of a 1 s interval between odor offset and face onset was also important in the present study. Our results suggest that shifts in face-evaluations were genuine priming effects evoked by the valence of the odors that carried over to the face evaluation phase, as opposed to affective responses to odors themselves.

Changes in ERP response to faces that occurred as a function of odor condition transpired during the late (>600 ms) and ultra-late (>900 ms) latency epochs. Indeed, the LPP is known to be sensitive to pleasant and unpleasant stimuli (Cacioppo et al., 1993; Cuthbert et al., 2000; Hajcak et al., 2006, 2007; Weinberg and Hajcak, 2010; Duval et al., 2013). In evaluative priming studies, the LPP component has typically been implicated in congruency effects (Herring et al., 2011). In one ERP study using pleasant odors and faces with pleasant and unpleasant expressions, Bensafi et al. (2002a) showed increased LPP amplitude for unpleasant faces preceded by pleasant odor primes, presumably due to the evaluative incongruence between the two. These findings provided initial evidence that the LPP reflects evaluative processes in a cross-modal sense, where olfactory stimuli influence processing of visual stimuli. Our results provide further evidence that cross-modal effects of odors on evaluations of faces may be reflected in late ERP components.

Significant changes in late ERP components observed in the present study included increased negativity in the right posterior- and temporal-parietal electrodes in the pleasant odor condition at 621 and 633 ms after face onset. This latency window corresponds with the mid-LPP observed in a recent study investigating ERPs in response to faces, where the authors suggested that this component is sensitive to the emotional content of faces (Duval et al., 2013). Since our study was the first to investigate effects of briefly presented pleasant and unpleasant odor-primes on ERPs in response to faces, the present findings are novel. However, Aguado et al. (2013) showed that positive targets elicited enhanced amplitudes relative to negative targets at parietal-occipital, fronto-central, and left temporal regions during the LPP. Further, Hermann et al. (2000) showed that appetitive conditioning with a pleasant odor elicited a stronger LPP (400−600 ms) relative to a no odor control. Taken together, these findings suggest that effects of positively-valenced stimuli may take precedence during late potential components, resulting in increased ERP amplitude. In the case of the present study, effects of the pleasant odor appeared to take hold during the late potential period, increasing ERP amplitude and corresponding with increased hedonic ratings of neutral faces. The larger LPP for faces preceded by pleasant odors may reflect the general influence of pleasant odors on evaluations of neutral stimuli. This furthers our understanding of the processes underlying odor-related evaluative shifts, and may have wider implications for understanding the neural basis of pleasant odor and cleanliness perception in both evolutionary and commercial contexts.

Significant effects of odor were also observed at 926 ms after face-onset, corresponding with the late-LPP observed in another study that investigated ERP response to faces (Duval et al., 2013). Results showed increased activation over lateral frontal-temporal electrodes in response to faces presented after a pleasant/unpleasant odor prime, in the left/right hemispheres, respectively. These findings support existing theories associating left hemisphere activity with processing of pleasant sensory stimuli, and right hemisphere activity with processing of unpleasant sensory stimuli (Tucker, 1981; Ahern and Schwartz, 1985; Mandal et al., 1991; Lane et al., 1997; Canli et al., 1998; Davidson, 1998; Lang et al., 1998). Hemispheric specialization of positive and negative affect has rarely been investigated in the field of olfaction specifically. However, the current finding corresponds with data showing that smelling pleasant and unpleasant odors increased activation in the left and right hemispheres, respectively (Henkin and Levy, 2001). The results also lend support for the suggestion that the right hemisphere is more efficient in decoding unpleasant affects induced by odors (Bensafi et al., 2002b), providing evidence that lateralization of valence processing applies to odors as well.

There was one significant and one marginally significant correlation between potential-amplitude data and baseline pleasantness ratings (taken before and after the task) at 926 ms. These suggested that participants who rated methylmercaptan as most unpleasant at baseline, showed greater activation in the left hemisphere during the late component in response to faces presented under that odor condition. Participants who perceived jasmine as more intense at baseline showed greater positive activation in the left hemisphere during the late component. However, correlations occurred with baseline ratings and during a long-latency component where there may have been a significant amount of variance. Therefore, interpretation of such correlations should be treated with caution. The lack of correlation between amplitudes and odor and face ratings suggests that strength of potentials may not precisely relate to odor-induced changes in hedonic ratings. Rather, a more general mechanism might be responsible for such effects.

One of the limitations of the present study was that, owing to a comparatively small number of face stimuli in each odor condition, effects of habituation on odor-induced changes in hedonic evaluation of faces remained unexplored. This effect was likely in the present study, as the interaction effects between experimental block and odors on face pleasantness ratings, and effect of block on odor intensity ratings pointed to a gradual decrease of hedonic effects of odors, especially in the unpleasant odor condition. Future studies involving single-trial analysis of ERPs and incorporating the time as an independent variable in statistical analysis should address this issue.

In summary, the present study used an exploratory ERP analysis to allow for the first investigation of the neural mechanisms underlying odor-induced changes in evaluations of faces. Results showed that effects of odors on face perception were reflected in late and ultra-late ERP components. Results suggest that effects of pleasant odors on face evaluation were specific to the late component. During the ultra-late component, effects of pleasant and unpleasant odors were distinguished in the left and right hemispheres, respectively. Further, our findings show that odors can alter hedonic evaluations of faces even when there is a slight temporal lag between presentation of odors and faces. Neutral faces presented after administration of a pleasant odor were rated significantly more pleasant than the same faces presented after administration of an unpleasant odor or clean air. It is likely that any positive or negative affect induced by previous pleasant or unpleasant odor stimulation carried over into the face evaluation phase.

## Conflict of Interest Statement

Authors Dr. A. Thomas and Dr. T. Giesbrecht are employed by Unilever. Unilever is interested in behavioral and cognitive effects of odors, as odors are natural ingredients of food and personal care products. However, none of the odors used in the present study are used commercially by Unilever. The other authors declare that the research was conducted in the absence of any commercial or financial relationships that could be construed as a potential conflict of interest.
